# Fluorinated Carbon Nanofibrous Aerogel Electrode Material Derived from Hydrofluoric Acid Treatment on Stabilized Polyacrylonitrile for High-Performance Supercapacitors

**DOI:** 10.3390/molecules30112282

**Published:** 2025-05-22

**Authors:** Victor Charles, Kingsford Asare, Md Faruque Hasan, Lifeng Zhang

**Affiliations:** Department of Nanoengineering, Joint School of Nanoscience and Nanoengineering, North Carolina A&T State University, Greensboro, NC 27401, USA

**Keywords:** supercapacitor, carbon electrode material, electrospinning, F doping, nanofibrous aerogel

## Abstract

Carbon nanofibrous materials from electrospinning are good candidate electrode materials for supercapacitor applications due to their straightforward processability, chemical stability, high porosity, and large surface area. In this research, a straightforward and effective way was revealed to significantly enhance the electrochemical performance of carbon nanofibrous electrode material from electrospinning of polyacrylonitrile (PAN). Fluorination of the electrospun carbon nanofibers (ECNF) was studied by comparing two types of hydrofluoric acid (HF) treatment, i.e., direct HF acid treatment on ECNF (Type I) vs. HF acid treatment on the stabilized PAN (Type II) followed by carbonization. The latter was found to be an advantageous way to introduce C-F bonds in the resultant carbon nanofibrous electrode material that contributed to pseudocapacitance. Furthermore, the Type II HF acid treatment demonstrated exciting synergistic effects with ECNF aerogel formation on carbon structure and porosity development and generated a superior fluorinated electrospun carbon nanofibrous aerogel (ECNA-F) electrode material for supercapacitor uses. The resultant ECNA-F electrode material demonstrated excellent electrochemical performance with great cyclic stability due to the large improvements in both pseudocapacitance and electrical double-layer capacitance. ECNA-F achieved a specific capacitance of 372 F/g at a current density of 0.5 A/g with 1 M H_2_SO_4_ electrolyte, and the device with ECNA-F and 1 M Na_2_SO_4_ electrolyte possessed an energy density of 29.1 Wh/kg at a power density of 275 W/kg. This study provided insight into developing high-performance and stable carbon nanofibrous electrode materials for supercapacitors.

## 1. Introduction

Over the years, there has been a fast increase in global energy demand, causing an over-dependence on fossil fuels and leaving a high carbon footprint [1,2,3]. To curtail this challenge, supercapacitors have been developed as electrical energy storage devices with characteristics such as fast charge/discharge, high power density, and good cyclic stability [4,5]. Supercapacitors can be mainly categorized into electrical double-layer capacitors (EDLCs) and pseudocapacitors. The separation of charges at the electrode/electrolyte interface is the mechanism of EDLC, making it similar to traditional electrolytic capacitors [6,7,8,9]. EDLCs exhibit a fast charge/discharge rate, but their specific capacitance and energy density are generally lower than those of pseudocapacitors. In the meantime, pseudocapacitors have relatively low cyclic stability. Thus, an appropriate balance of EDLC capacitance with pseudocapacitance is desired for developing supercapacitor electrode materials with comprehensive electrochemical performance.

Carbon materials have been widely considered efficient electrode materials for supercapacitors due to their good conductivity, stability, and mechanical properties. Out of various ways of making carbon materials, the electrospinning method has gained much attention due to its simplicity in producing submicron and nanometer-scale carbon fibers, which demonstrated the virtues of high surface area and structural stability [10]. To further improve the electrochemical performance of electrospun carbon nanofibers, highly porous sponge-like materials with large volume-to-mass ratio, i.e., carbon nanofibrous aerogel materials, have been reported in recent years [11,12]. The high porosity and wide range of pore size distribution, like micropores and mesopores that are present in carbon nanofibrous aerogels, make them suitable as electrode materials for supercapacitors.

Heteroatom doping is another effective method to improve the supercapacitor performance of carbon electrode materials by improving charge redistribution in carbon lattice [13]. Among a number of heteroatoms used in various works, F doping is attractive and can improve the specific capacitance of carbon electrode materials by the formation of semi-ionic C-F bonds and a change in the chemical environment of carbon in the carbon lattice [14]. Notably, carbon materials with a high degree of fluorination could result in poor electrical conductivity because the strong electron withdrawal capability of F atoms causes the disappearance of many π-conjugated bonds in carbon when C-F bonds are formed [15]. Therefore, an appropriate way for carbon fluorination is needed for carbon electrode materials to achieve high electrochemical performance.

In this research, we first investigated the fluorination of carbon nanofibers from electrospinning polyacrylonitrile (PAN) [16]. Acid treatment has been popularly used to introduce surface acidic functional groups to carbon fibers [17]. Among the various acids used for carbon surface treatment, hydrofluoric acid (HF) is a good carbon etchant that increases surface area and fluorinates carbon materials [18]. Herein, we studied two types of HF acid treatment on the electrospun carbon nanofibers (ECNF). In Type I, HF acid treatment was performed on ECNF directly. In Type II, HF acid treatment was performed on the stabilized electrospun PAN nanofibers (an intermediate of ECNF), followed by carbonization. After determining a better method for fluorinating ECNF, we applied it to fluorinate electrospun carbon nanofibrous aerogel (ECNA) material and evaluated its electrochemical performance for supercapacitor uses. By combining the Type II HF acid treatment and aerogel formation, we realized a superior supercapacitor electrode material, i.e., fluorinated electrospun carbon nanofibrous aerogel material (ECNA-F) through simultaneous improvements in pseudocapacitance and EDLC capacitance. This research shed light on the design of high-performance carbon electrode materials for supercapacitors.

## 2. Results and Discussion

### 2.1. Morphology

**Figure 1:**
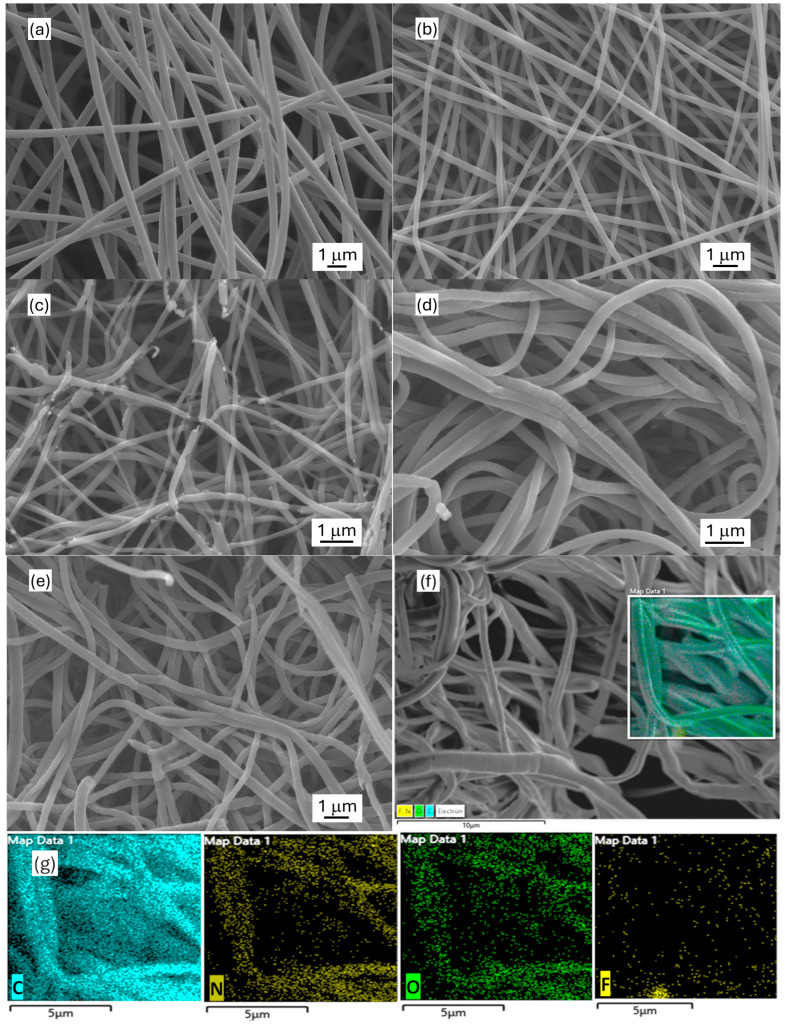
SEM images of electrospun carbon nanofibrous electrode materials: (**a**) ECNF; (**b**) F-ECNF; (**c**) ECNF-F; (**d**) ECNA; (**e**) ECNA-F; (**f**) ECNA-F for EDX mapping; and (**g**) respective C, N, O, F EDX mapping of ECNA-F.

The morphology of the synthesized carbon nanofibrous electrode materials was characterized by SEM (Figure 1). The obtained electrospun carbon nanofiber (ECNF) mat comprised uniform cylindrically shaped nanofibers with an average size of ~450 nm. The fluorinated ECNF using Type I fluorination (F-ECNF) showed a smaller average fiber size of ~250 nm. The fluorinated ECNF using Type II fluorination (ECNF-F) exhibited more broken fibers with a further reduced average size of ~180 nm and a broader range of sizes. The electrospun carbon nanofibrous aerogel (ECNA) bore densely packed carbon nanofibers with an average size of ~300 nm, which could be caused by freeze-drying and crosslinking. The fluorinated ECNA (ECNA-F) possessed a smaller average fiber size of ~230 nm and more broken fibers. EDX elemental mapping was performed on ECNA-F to check the distribution of C, N, O, and F elements. The results confirmed the existence of C, N, O, and F elements in ECNA-F and showed even distributions of C, N, O, and F elements along the nanofibers, which also proved successful fluorination.

### 2.2. Structure

**Figure 2:**
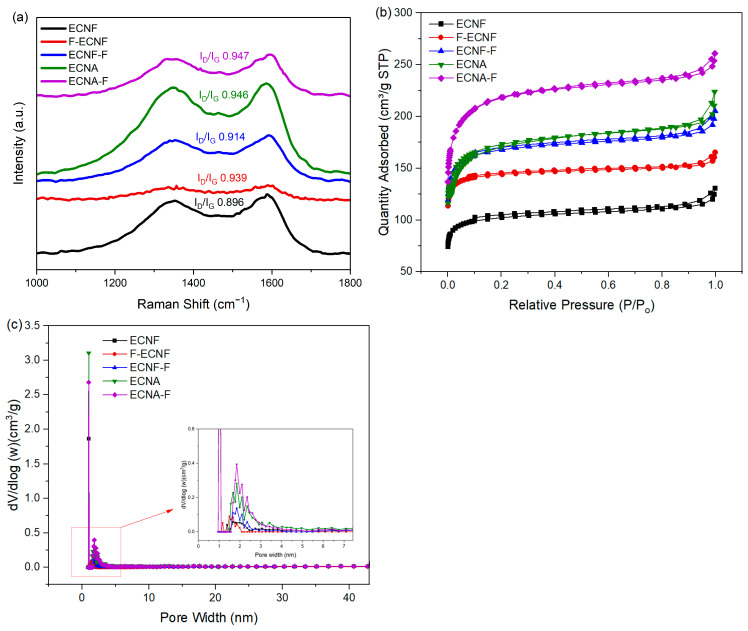
Raman spectra (**a**), N_2_ adsorption–desorption isotherms (**b**), and pore size distribution (**c**) of ECNF, F-ECNF, ECNF-F, ENCA, and ENCA-F nanofibrous electrode materials.

Raman spectroscopy with D (1348 cm^−1^) and G (1588 cm^−1^) bands were used to study the structural variation and defects of the carbon nanofibrous electrode materials. The G band represents sp^2^ hybridized carbon atoms that are associated with ordered carbon structure (graphitic structure), while the D band represents sp^3^ hybridized carbon atoms that are associated with defect sites and disordered carbon structure. The intensity ratio of the D band to the G band is generally used to explain the magnitude of disorder and defects in carbon materials. The I_D_/I_G_ ratio of the carbon nanofibrous samples in this research (Figure 2a) indicated that direct HF treatment (Type I), as well as aerogel processing, largely increased the defects in carbon nanofibers. Compared to Type I, the HF acid treatment through Type II significantly reduced the defects in carbon nanofibers, but no significant change was observed for the Type II HF acid treatment on the carbon nanofibrous aerogel material.

The N_2_ adsorption/desorption isotherms of the prepared carbon nanofibrous electrode materials are presented in Figure 2b. All the samples exhibited type IV isotherms with H3 hysteresis at 0.1–1.0 relative pressure range. This can be attributed to the effect of capillary condensation, indicating the presence of mesopores in the prepared samples [19]. BET tests were carried out to determine the specific surface area and total pore volume of the prepared carbon nanofibrous electrode materials. As shown in Table 1, the HF acid treatment and aerogel formation improved the specific surface area and pore volume of corresponding nanofibrous electrode materials. The Type II HF acid treatment on ECNF resulted in an increase in micropores, mesopores, and macropores. In contrast, Type I HF treatment on ECNF only showed an increase in micropores with reduced mesopores and macropores. Taking advantage of the Type II HF acid treatment, the fluorinated carbon nanofibrous aerogel electrode material (ECNA-F) gave the highest specific surface area of 703 m^2^/g and the largest pore volumes including micropore volume of 0.0181 cm^3^/g, mesopore volume of 0.0568 cm^3^/g, macropore volume of 0.0198 cm^3^/g, and total pore volume of 0.0947 cm^3^/g. The aerogel formation and Type II HF acid treatment led to a synergistic effect in improving micropores, mesopores, and macropores of the resultant carbon nanofibrous electrode material (Figure 2c).

**Figure 3:**
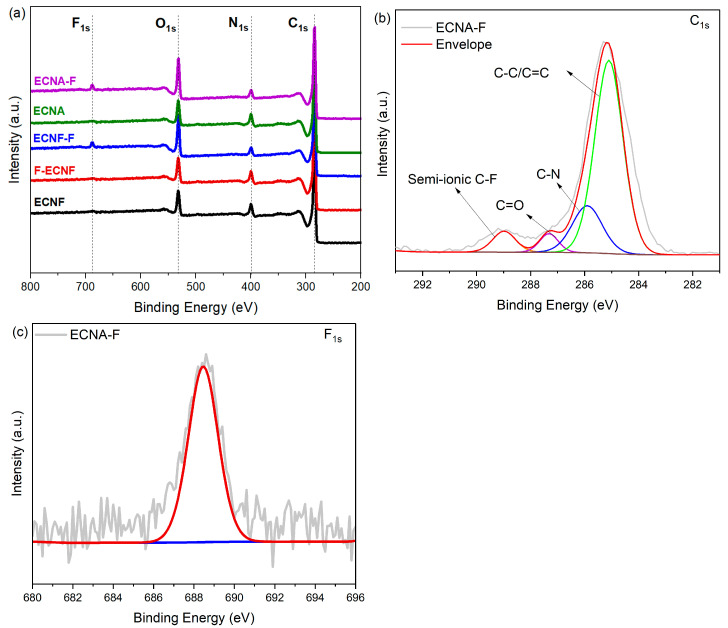
XPS survey spectra of ECNF, F-ECNF, ECNF-F, ECNA, ECNA-F (**a**), high-resolution C_1s_ spectrum of ECNA-F (**b**), and high-resolution F_1s_ spectrum of ECNA-F (**c**).

X-ray photoelectron spectroscopy (XPS) was used to characterize the surface of the prepared carbon nanofibrous electrode materials. According to the C, N, O, and F XPS survey spectra of the carbon nanofibrous electrode materials shown in Figure 3a, the ECNF material that went through Type I HF acid treatment (F-ECNF) did not show a significant F_1s_ peak, indicating that the Type I HF acid treatment, i.e., direct HF acid surface treatment of the carbon nanofibers, did not result in effective fluorination of the ECNF. After the ECNF and ECNA aerogel materials went through Type II HF acid treatment, the resultant ECNF-F and ECNA-F exhibited significant F_1s_ peak, indicating Type II HF acid treatment is a compelling way to fluorinate carbon nanofibers. The high-resolution XPS C_1s_ spectrum of ECNA-F could be deconvoluted into four peaks at 284.8, 286.1, 287.1, and 288.8 eV (Figure 3b), which can be assigned to C-C/C=C, C-N, C=O, and C-F bonds [20,21], respectively, confirming the effective fluorination. The high-resolution F_1s_ spectrum of ECNA-F also showed a peak at 688.5 eV (Figure 3c), a verification of C-F bond formation after Type II HF acid treatment.

### 2.3. Electrochemical Performance

The electrochemical performance of the prepared carbon nanofibrous electrode materials was evaluated by cyclic voltammetry (CV), galvanostatic charge/discharge (GCD), and electrochemical impedance spectra (EIS) through a symmetric two-electrode cell.

**Figure 4:**
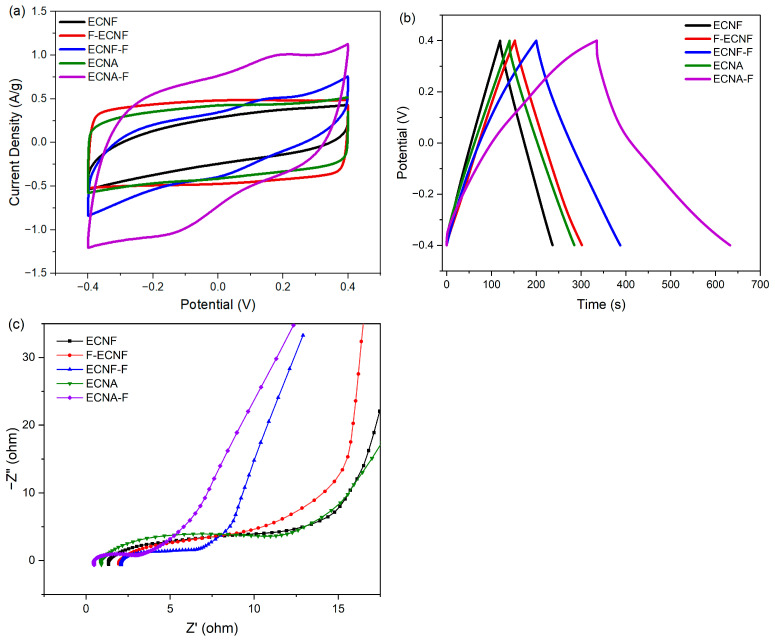
Cyclic voltammetry (CV) curves at a scan rate of 5 mV/s (**a**), galvanostatic charge/discharge (GCD) profiles at a current density of 0.5 A/g (**b**), and Nyquist plots (**c**) of the prepared carbon nanofibrous electrode materials.

As shown in Figure 4a, the CV curve of ECNF was quasi-rectangular, demonstrating an electrochemical double-layer capacitance (EDLC capacitance). F-ECNF and ECNF-F showed different CV behaviors. The CV curve of F-ECNF exhibited a typical rectangular shape, implying an EDLC capacitance, and had a larger enclosed area compared to that of ECNF. For comparison, ECNF-F showed a quasi-rectangular CV curve with a larger area than that of ECNF, indicating the presence of both EDLC and pseudocapacitance. As for the nanofibrous aerogel ECNA, the enclosed area of its CV curve was larger than that of ECNF and showed a typical rectangular shape, while the CV curve of ECNA-F showed a quasi-rectangular shape with the largest enclosed CV area among all the studied electrode materials, suggesting both EDLC and pseudocapacitance.

According to the GCD tests of the prepared carbon nanofibrous electrode materials at a current density of 0.5 A/g with 1 M H_2_SO_4_ electrolyte (Figure 4b), ECNF showed a specific capacitance of 141 F/g. Carrying out Type I HF acid treatment achieved a specific capacitance of 187 F/g (F-ECNF), while Type II HF acid treatment further increased the specific capacitance to 235 F/g (ECNF-F). Nanofibrous aerogel formation improved the specific capacitance to 180 F/g (ECNA) with respect to ECNF. The Type II HF acid treatment on ECNA significantly increased the corresponding specific capacitance to 372 F/g (ECNA-F). Notably, GCD curves of ECNF-F and ECNA-F exhibited a deviation from an ideal triangle shape, implying pseudocapacitive behavior.

EIS test was performed to analyze the structure-performance relationship of the prepared carbon nanofibrous electrode materials. According to the Nyquist plots (Figure 4c), ECNA-F showed the smallest electrolyte resistance (the smallest semi-circle in the high-frequency range) and the smallest electrode resistance (the smallest intercept at the axis of the real part of complex impedance), which is consistent with its best electrochemical performance.

**Figure 5:**
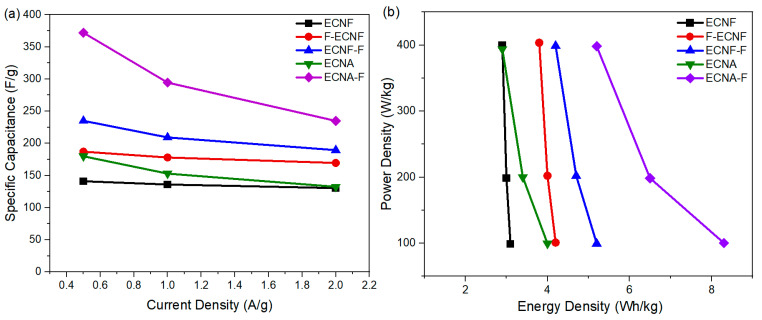
Specific capacitances from GCD at various current densities (**a**) and power density vs. energy density (**b**) of the cells with the prepared carbon nanofibrous electrode materials.

The dependence of specific capacitance of respective carbon nanofibrous electrode materials on current densities is shown in Figure 5a. By increasing the current density from 0.5 A/g to 2 A/g, the specific capacitance of all the carbon nanofibrous electrode materials was reduced. The specific capacitance of ECNF decreased from 141 F/g to 131 F/g when the current density increased from 0.5 A/g to 2 A/g, retaining 92% of its initial specific capacitance. By contrast, the specific capacitance of ECNA-F decreased to 235 F/g at a current density of 2 A/g, retaining 63% of its initial specific capacitance. The Type II HF acid treatment, as well as aerogel formation, resulted in more sensitivity to the current density. This phenomenon could be attributed to the large micropore volumes of ECNA and ECNA-F. Under low current density, electrolyte ions have enough time to access the micropores, while under high current density, the electrolyte ions would not have enough time to access the micropores, and the electrode material thus showed more reduced specific capacitance.

The practical use of the prepared carbon nanofibrous electrode materials was evaluated by their corresponding cell energy densities and power densities (Figure 5b). The cell energy density with ECNF in 1 M H_2_SO_4_ electrolyte dropped from 3.1 Wh/kg at a power density of 98.8 W/kg to 2.9 Wh/kg at a power density of 400 W/kg. Meanwhile, the cell energy density with the fluorinated carbon nanofibrous aerogel electrode material through Type II HF acid treatment (ECNA-F) dropped from 8.3 Wh/kg at a power density of 100.4 W/kg to 5.2 Wh/kg at a power density of 398.3 W/kg.

**Figure 6:**
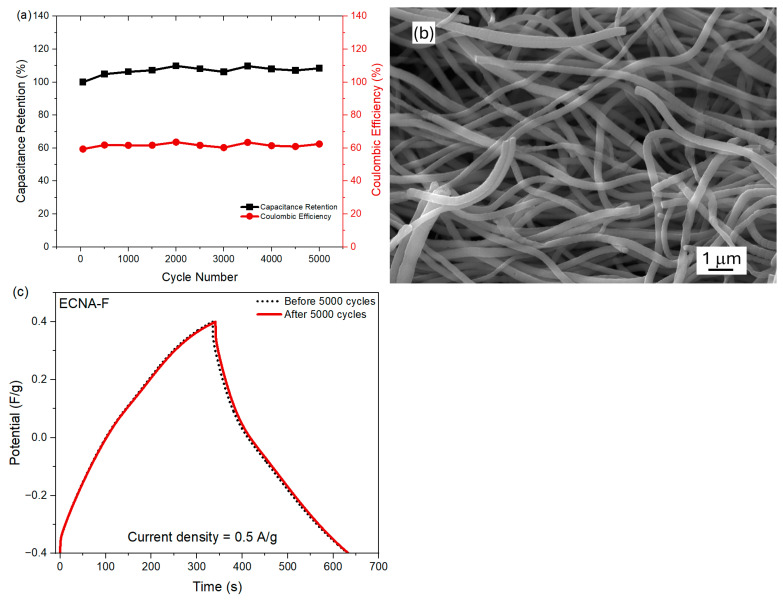
Cyclic stability test of the cell with ECNA-F at a current density of 10 A/g (**a**), SEM image of ECNA-F after 5000 charge/discharge cycles (**b**), and GCD curves of the cell with ECNA-F before and after 5000 charge/discharge cycles (**c**).

Furthermore, the cyclic stability of fluorinated carbon nanofibrous aerogel electrode material (ECNA-F) in the two-electrode cell was measured at a current density of 10 A/g (Figure 6a). ECNA-F exhibited outstanding stability in the cell by showing no loss in capacitance as well as maintaining a coulombic efficiency of ~62% after 5000 cycles, indicating its long-term stability as electrode material. The relatively low Coulomb efficiency could be attributed to the large micropore volume of the electrode material. During the charging time, it took more time for electrolyte ions to transport and access all the available surfaces, including micropores of the electrode material, while the electrolyte ions in micropores might not be fully recovered during the discharge process. The SEM examination revealed that there was no significant change in the morphology of ECNA-F after 5000 charge/discharge cycles (Figure 6b). Additionally, GCD tests of the cell with ECNA-F at 0.5 A/g before and after 5000 charge/discharge cycles demonstrated a capacitance retention of 98% (Figure 6c).

**Figure 7:**
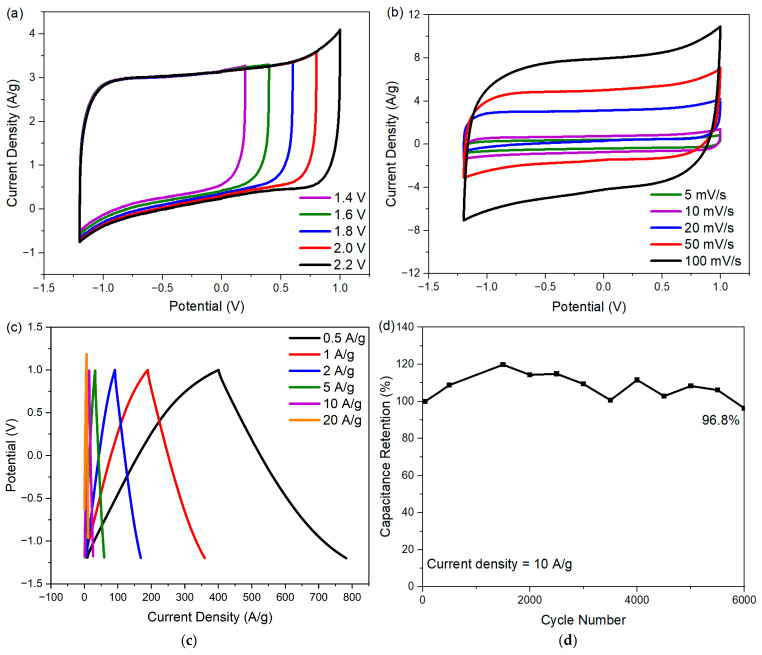
Electrochemical performance of ECNA-F in 1 M Na_2_SO_4_ electrolyte: (**a**) CV curves in different potential windows with a scan rate of 20 mV/s; (**b**) CV curves at different scan rates in the potential window from −1.2 to 1.0 V; (**c**) GCD curves at different current densities; and (**d**) capacitance retention up to 6000 cycles.

To improve the energy density of the cell with ECNA-F electrode material, Na_2_SO_4_ electrolyte was used. The choice of Na_2_SO_4_ is due to the strong solvation of both Na^+^ and SO_4_^2−^ ions in an aqueous medium, giving it the capability to be used with a wider potential window [22,23]. The CV curves of the cell with ECNA-F at different potential windows (1.4 to 2.2 V) are shown in Figure 7a. The ECNA-F electrode material could reach a 2.2 V potential window without distortion in the rectangular shape of its CV curve, indicating no oxygen and hydrogen evolution. According to the CV tests of ECNA-F at scan rates from 5 to 100 mV/s within a potential window of −1.2 to 1.0 V (Figure 7b), the CV curves retained their quasi-rectangular shape even at high scan rates, indicating outstanding electrochemical performance. The GCD curves of ECNA-F (Figure 7c) demonstrated perfect symmetry, implying good reversibility. The ECNA-F electrode material possessed a specific capacitance of 173 F/g at a current density of 0.5 A/g with 1 M Na_2_SO_4_ electrolyte, and 54.5% of the initial capacitance was retained when the current density was increased to 20 A/g. A 96.8% cell capacitance retention was achieved after 6000 charge/discharge cycles at 10 A/g current density with ECNA-F electrode material and 1 M Na_2_SO_4_ electrolyte (Figure 7d). The relatively large fluctuation of specific capacitance during the cycling test could be attributed to the relatively slow charge transfer/ion migration in the porous electrode material and consequent variations in charge/discharge responses due to the relatively large hydration sphere radius and reduced ionic mobility and molar ionic conductivity of Na^+^ ions compared to H^+^ ions [24]. The cell with ECNA-F electrode material and 1 M Na_2_SO_4_ electrolyte achieved an energy density of 29.1 Wh/kg at a power density of 275 W/kg.

### 2.4. Discussion of Electrochemical Performance

Among all the electrospun carbon nanofibrous electrode materials studied, the aerogel formation, as well as the type II HF acid treatment, effectively improved the electrochemical performance of the resultant electrospun carbon nanofibrous electrode materials.

(1) Aerogel formation (ECNF vs. ECNA)

Compared to ECNF, ECNA possessed much more defects in its carbon nanofibers, evidenced by the I_D_/I_G_ ratio from Raman spectra (increased from 0.896 for ECNF to 0.945 for ECNA). In the meantime, the micropore volume, mesopore volume, macropore volume, and total pore volume increased by 145%, 71%, 72%, and 78%, respectively, after aerogel formation. All these structural changes provided more available surfaces for the electrode material to store charges. As a result, the specific capacitance of the respective electrode materials improved from 141 F/g for ECNF to 180 F/g for ECNA (~28% increase).

(2) HF acid treatment (F-ECNF vs. ECNF-F)

Based on the structural and electrochemical evaluation, Type II HF acid treatment overwhelmed Type I HF acid treatment for the purpose of fluorination, as evidenced by the XPS results (Figure 3) and elemental mapping (Figure 1). The fluorination of ECNF through Type II HF acid treatment resulted in a significant amount of semi-ionic C-F bonds, which induced significant pseudocapacitance as evidenced by CV and GCD profiles (Figure 4a,b). In comparison with Type II HF acid treatment, Type I HF acid treatment only etched carbon without forming appreciable C-F bonds.

According to Trasatti analysis [25,26], the maximum total capacitance and the maximum EDLC capacitance were estimated. The difference between the total capacitance and the EDLC capacitance could give the maximum pseudocapacitance. The EDLC capacitance and pseudocapacitance contributions of F-ECNF were 78% and 22%, respectively. For ECNF-F, the capacitance contributions were 35% for EDLC and 65% for pseudocapacitance. These results indicated the more important role of pseudocapacitance in ECNF-F, which was caused by the effective fluorination of ECNF through type II HF acid treatment and consequent semi-ionic C-F bond formation. In contrast, F-ECNF showed lower pseudocapacitance contribution, which is in agreement with the lower fluorination effectiveness from Type I HF acid treatment.

(3) Synergistic effects from the combination of Type II HF acid treatment and aerogel formation

The superior electrochemical performance of ECNA-F electrode material indicated the exciting synergistic effect from the combination of aerogel formation and Type II HF acid treatment. ECNA-F not only possessed the highest specific surface area (113% increase with respect to ECNF) and the largest pore volumes (300% increase in micropore volume, 98% increase in mesopore volume, 25% increase in macropore volume, and 93% increase in total pore volume with respect to ECNF) among all the studied carbon nanofibrous electrode materials but also the greatest fluorination. The aerogel structure of ECNA-F with the simultaneously largest micropore, mesopore, and total volume could facilitate the transportation of electrolyte ions into all available surfaces for charge storage, including those normally hard-to-access micropores. Our previous research indicated that mesopores could assist electrolyte ions to access micropores [10]. The largest specific surface area of ECNA-F enabled its largest EDLC, while the most F heteroatoms in ECNA-F allowed the highest pseudocapacitance via surface faradaic redox reactions caused by the semi-ionic bonds of C-F in which the carbon atoms from C-F could become an acidic character and behave as an electron acceptor so that the oxidation/reduction can occur [27]. Consequently, ECNA-F exhibited the smallest electrolyte resistance, the smallest electrode resistance, and the highest specific capacitance of 372 F/g at 0.5 A/g current density from both EDLC capacitance and pseudocapacitance. It is noteworthy that ECNA-F also showed outstanding capacitance retention after 5000 charge/discharge cycles and exhibited even higher specific capacitance than that at the beginning. This result could be attributed to additional pore openings in the process of charge/discharge cycles caused by the diffusion of electrolyte ions into micropores, leading to more charge storage sites. Its steady coulombic efficiency suggested a stable long-term performance.

It is noteworthy that our ECNA-F electrode materials outperformed the previously reported F-doped carbon aerogel electrode materials [28,29,30] as well as undoped carbon aerogel electrode materials [31,32] for supercapacitor uses in terms of specific capacitance, rate capability, and cycling stability, which proved the advances in this research.

## 3. Materials and Methods

### 3.1. Materials

Polyacrylonitrile (PAN, Mw = 150,000), N, N-dimethylformamide (DMF), tert-butanol, 1,4 Benzoxazin, sulfuric acid (H_2_SO_4_, 98%), hydrofluoric acid (HF), and sodium sulfate (Na_2_SO_4_) were purchased from Sigma-Aldrich. All chemicals were purchased without any further purification.

### 3.2. Preparation of Electrospun PAN Nanofibers

PAN and benzoxazine were dissolved in DMF at 50 °C with magnetic stirring for 2 h to make a DMF solution containing 10 wt.% PAN and 1 wt.% benzoxazine as an aerogel crosslinking agent. The solution was further stirred continuously overnight to obtain a homogeneous spin dope. The electrospinning was conducted in a SpinBox electrospinning unit (Nanoscience Instruments, Phoenix, AZ, USA) with 15 kV voltage, 1 mL/h solution feed rate, 17 cm collecting distance, and 460 rpm rotation speed of the collecting drum.

### 3.3. Preparation of Electrospun Carbon Nanofibers

To prepare electrospun carbon nanofibers, the electrospun PAN nanofibrous mat was first stabilized in air at 280 °C for 6 h with a heating rate of 1 °C/min and then carbonized at 900 °C for 1 h with a heating rate of 5 °C/min in a Carbolite HTF 1800 box furnace under a nitrogen atmosphere with a continuous N_2_ gas flow of 100 mL/min. The obtained carbon nanofibers were denoted as ECNF.

### 3.4. Preparation of Fluorinated Carbon Nanofibers

Two types of HF acid treatment were performed to make fluorinated ECNF. In Type I treatment, ECNF from stabilization and carbonization of electrospun PAN, as described in Section 2.3, were directly treated with 20 wt.% HF acid at room temperature for 12 h followed by being thoroughly washed with DI water and dried in an oven at 60 °C for another 12 h. The obtained fluorinated carbon nanofibrous material was denoted as F-ECNF. In Type II treatment, the stabilized electrospun PAN nanofibers were immersed in 20 wt.% HF acid at room temperature for 12 h. Next, the treated nanofibers were taken out of HF acid, washed with DI water thoroughly, and dried in an oven at 60 °C for 12 h. The dried nanofibers were then carbonized under the same condition as described in Section 2.3. The acquired fluorinated carbon nanofibrous material was denoted as ECNF-F.

### 3.5. Preparation of Fluorinated Carbon Nanofibrous Aerogel

**Figure 8:**
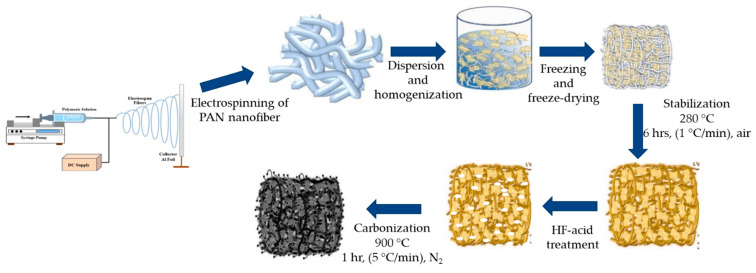
Schematic diagram of the preparation of fluorinated carbon nanofibrous aerogel (ECNA-F).

To make carbon nanofibrous aerogel material, the electrospun PAN nanofibrous mat was first cut into small pieces (1 cm × 1 cm) and homogenized in water with ethanol/tert-butanol added by using a T25 homogenizer. Then 30 mL of the homogenized aqueous dispersion with 0.3 g of nanofibers was placed in a freezer overnight, followed by being freeze-dried for 48 h. The obtained PAN nanofibrous aerogel was stabilized and carbonized following the procedure described in Section 2.3. The resultant carbon nanofibrous aerogel was denoted as ECNA. The fluorinated ECNA was prepared through Type II fluorination and denoted as ECNA-F. The preparation procedure is illustrated in Figure 8.

### 3.6. Electrochemical Properties

A symmetric two-electrode cell setup was used to analyze the electrochemical properties of the prepared carbon nanofibrous electrode materials. Two pieces of equal-mass respective carbon nanofibrous material were assembled in the cell, separated by a Whatman grade 6 qualitative cellulose filter paper (standard grade, nominal thickness 180 μm, pore size 3 μm) as an economic semi-permeable membrane to allow electrolyte diffusion and prevent short circuit, and soaked in 1 M H_2_SO_4_ electrolyte. A CHI 660E electrochemical workstation was used to conduct electrochemical analysis of all the prepared samples.

The specific capacitance of the respective electrode material in the cyclic voltammetry test was calculated using Equation (1).

(1) $$C=\frac{1}{mv\left(V_{t}-V_{0}\right)}\;\int_{V_{0}}^{V_{t}}I\left(V\right)dV$$

where *m* is the mass of the electrode material (g), *I* is the current (A), *v* is the scan rate (mV/s), and *V*_0_ and *V_t_* are the lower and upper potential limits of the potential window used.

The specific capacitance of the respective electrode material in the discharge part of galvanostatic charge/discharge (GCD) curves was calculated using Equation (2).

(2) $$C=\frac{2I∆t}{m∆V}$$

where *I* represents the discharge current (A), ∆*t* is the discharge time (s), *m* represents the mass of a single electrode material (g), and ∆*V* represents the potential window.

The energy density (*E*, Wh/kg) of the device with respective electrode material was calculated by Equation (3).

(3) $$E=\frac{1}{8\times 3.6}\times C\times {\left(∆V\right)}^{2}$$

where *C* represents the specific capacitance of the electrode material from the GCD test, and ∆*V* represents the potential window.

The power density P of the device was obtained using Equation (4).

(4) $$P=\frac{E\times 3600}{t}$$

where *E* is the energy density (Wh/kg), and *t* is the discharge time (s).

## 4. Conclusions

In this research, a superior carbon electrode material, i.e., fluorinated electrospun carbon nanofibrous aerogel (ECNA-F), was successfully prepared by hydrofluoric acid (HF) acid treatment of stabilized polyacrylonitrile nanofibrous aerogel material (Type II HF acid treatment) followed by carbonization. Compared to the direct HF acid treatment on electrospun carbon nanofibrous material (ECNF), i.e., Type I HF acid treatment, Type II HF acid treatment not only generated a significant degree of fluorination in the resultant carbon nanofibrous materials, which enhanced its pseudocapacitance, but also facilitated micropore, mesopore, and macropore development in ECNF along with aerogel processing, which enhanced its electrical double-layer capacitance. The exciting synergistic effects from the combination of aerogel formation and Type II HF acid treatment resulted in the outstanding electrochemical performance of ECNA-F, including a specific capacitance of 372 F/g at 0.5 A/g current density with 1 M H_2_SO_4_ electrolyte, an increase by 164% with respect to that of unmodified ECNF, and no loss in capacitance after 5000 charge/discharge cycles. The device with ECNA-F electrode material and 1 M Na_2_SO_4_ electrolyte achieved an energy density of 29.1 Wh/kg at a power density of 275 W/kg in the company of a wide potential window of −1.2 to 1.0 V. The findings of this study revealed a simple and effective way to enhance the electrochemical performance of electrospun carbon nanofibers as standalone electrode material for supercapacitor applications.

## Figures and Tables

**Table 1:** BET surface area and pore volume of the carbon nanofibrous electrode materials.

Sample Name	BET Surface Area	Average Pore Size	V_micro_ (cm^3^/g)	V_meso_ (cm^3^/g)	V_macro_ (cm^3^/g)	V_total_ (cm^3^/g)
ECNF	329	5.4	0.00453	0.0287	0.0158	0.0491
F-ECNF	469	5.1	0.00474	0.0207	0.0131	0.0338
ECNF-F	539	4.98	0.00795	0.0399	0.0207	0.0685
ECNA	559	4.77	0.0111	0.0491	0.0271	0.0873
ECNA-F	703	3.92	0.0181	0.0568	0.0198	0.0947

## Data Availability

The original contributions presented in this study are included in the article. Further inquiries can be directed to the corresponding author(s).
